# Supplementary material for: Full-Chain FeCl3 Catalyzation Is Sufficient to Boost Cellulase Secretion and Cellulosic Ethanol along with Valorized Supercapacitor and Biosorbent Using Desirable Corn Stalk
Source: Molecules. 2023 Feb 22;28(5):2060. doi: 10.3390/molecules28052060 (PMC10004197; doi:10.3390/molecules28052060)
Supplement: Supplementary file 1 [file molecules-28-02060-s001.zip › molecules-2210199-supplementary.pptx]

## Slide 1
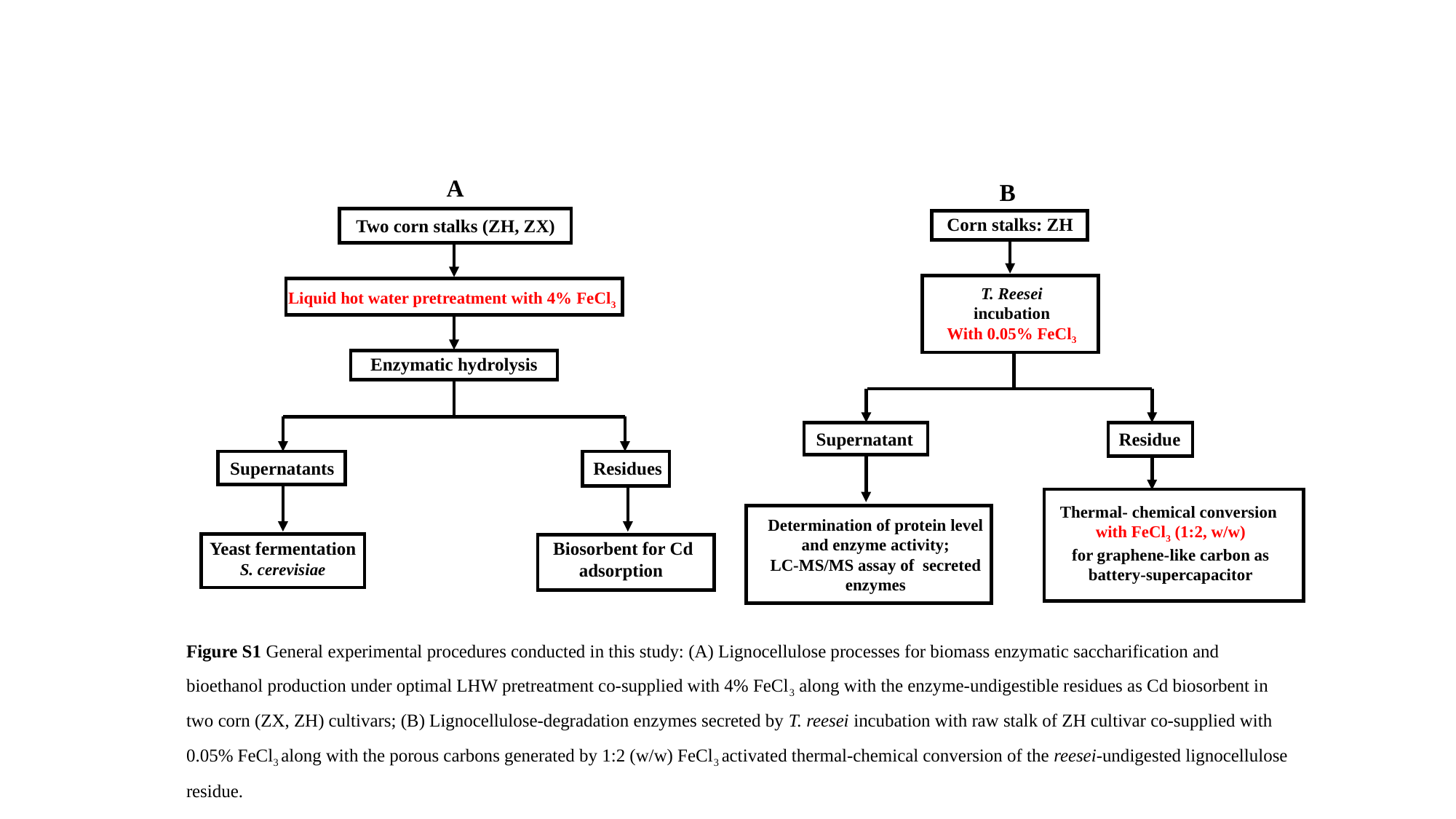

A
B
Corn stalks: ZH
T. Reesei
incubation
With 0.05% FeCl3
Supernatant
Residue
Thermal- chemical conversion
with FeCl3 (1:2, w/w)
for graphene-like carbon as battery-supercapacitor
Determination of protein level and enzyme activity;
LC-MS/MS assay of secreted enzymes
Two corn stalks (ZH, ZX)
Liquid hot water pretreatment with 4% FeCl3
Enzymatic hydrolysis
Supernatants
Residues
Yeast fermentation
S. cerevisiae
Biosorbent for Cd adsorption
Figure S1 General experimental procedures conducted in this study: (A) Lignocellulose processes for biomass enzymatic saccharification and bioethanol production under optimal LHW pretreatment co-supplied with 4% FeCl3 along with the enzyme-undigestible residues as Cd biosorbent in two corn (ZX, ZH) cultivars; (B) Lignocellulose-degradation enzymes secreted by T. reesei incubation with raw stalk of ZH cultivar co-supplied with 0.05% FeCl3 along with the porous carbons generated by 1:2 (w/w) FeCl3 activated thermal-chemical conversion of the reesei-undigested lignocellulose residue.

## Slide 2
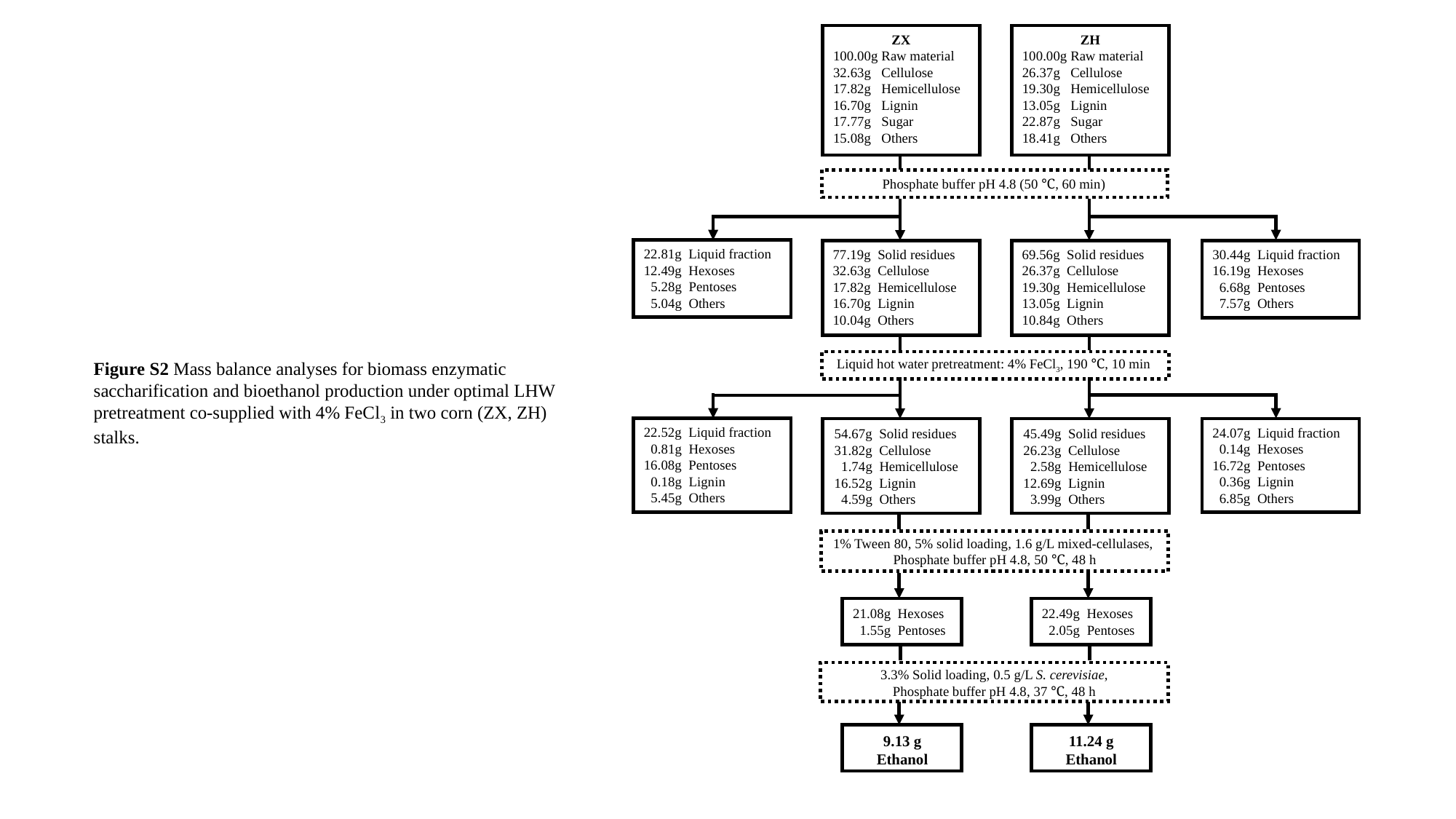

ZX
100.00g Raw material
32.63g Cellulose
17.82g Hemicellulose
16.70g Lignin
17.77g Sugar
15.08g Others
ZH
100.00g Raw material
26.37g Cellulose
19.30g Hemicellulose
13.05g Lignin
22.87g Sugar
18.41g Others
Phosphate buffer pH 4.8 (50 ℃, 60 min)
22.81g Liquid fraction
12.49g Hexoses
 5.28g Pentoses
 5.04g Others
77.19g Solid residues
32.63g Cellulose
17.82g Hemicellulose
16.70g Lignin
10.04g Others
69.56g Solid residues
26.37g Cellulose
19.30g Hemicellulose
13.05g Lignin
10.84g Others
30.44g Liquid fraction
16.19g Hexoses
 6.68g Pentoses
 7.57g Others
Liquid hot water pretreatment: 4% FeCl3, 190 ℃, 10 min
22.52g Liquid fraction
 0.81g Hexoses
16.08g Pentoses
 0.18g Lignin
 5.45g Others
24.07g Liquid fraction
 0.14g Hexoses
16.72g Pentoses
 0.36g Lignin
 6.85g Others
54.67g Solid residues
31.82g Cellulose
 1.74g Hemicellulose
16.52g Lignin
 4.59g Others
45.49g Solid residues
26.23g Cellulose
 2.58g Hemicellulose
12.69g Lignin
 3.99g Others
1% Tween 80, 5% solid loading, 1.6 g/L mixed-cellulases,
Phosphate buffer pH 4.8, 50 ℃, 48 h
21.08g Hexoses
 1.55g Pentoses
22.49g Hexoses
 2.05g Pentoses
3.3% Solid loading, 0.5 g/L S. cerevisiae,
Phosphate buffer pH 4.8, 37 ℃, 48 h
9.13 g
Ethanol
11.24 g
Ethanol
Figure S2 Mass balance analyses for biomass enzymatic saccharification and bioethanol production under optimal LHW pretreatment co-supplied with 4% FeCl3 in two corn (ZX, ZH) stalks.

## Slide 3
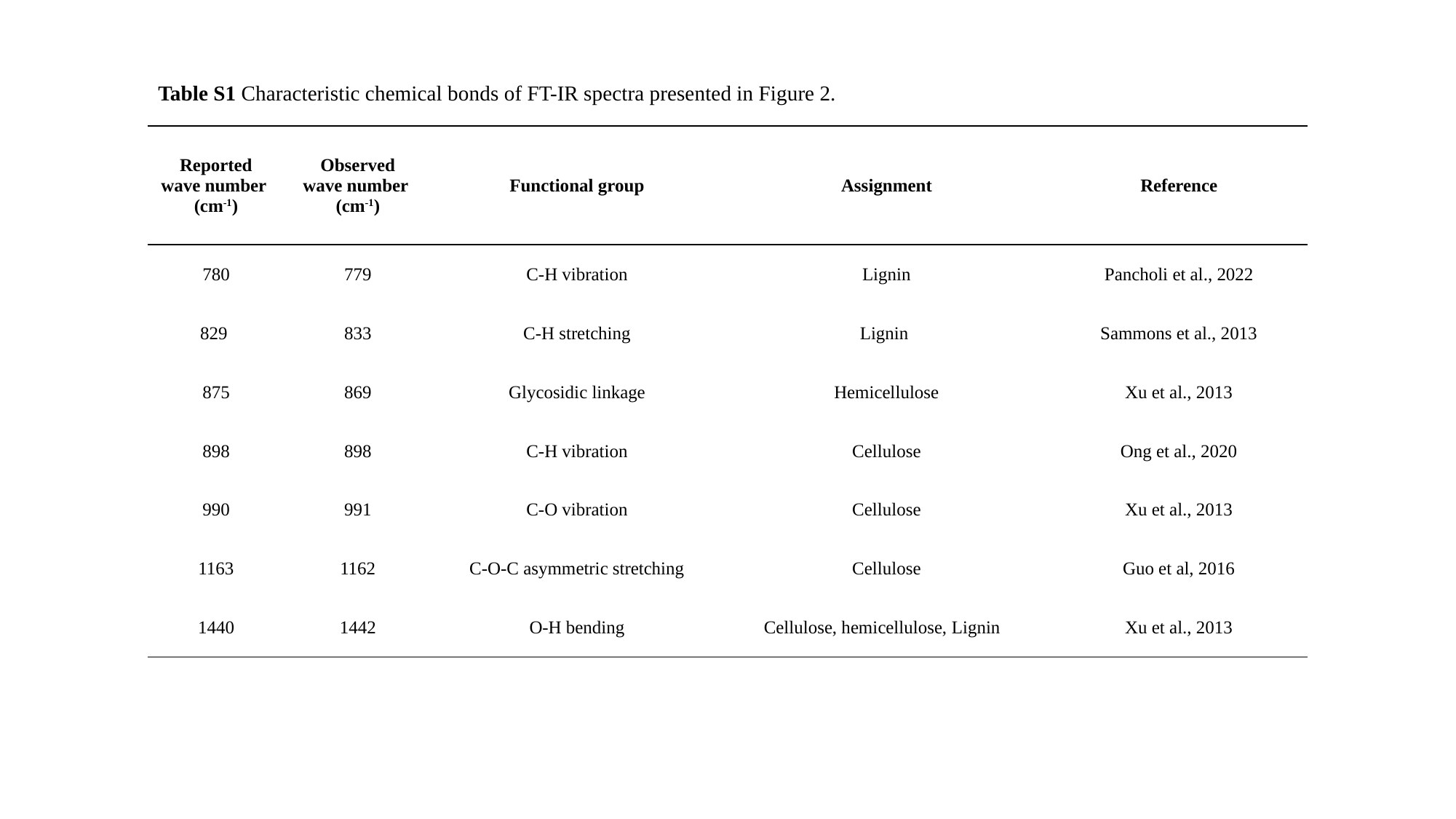

Table S1 Characteristic chemical bonds of FT-IR spectra presented in Figure 2.
| Reported wave number (cm-1) | Observed wave number (cm-1) | Functional group | Assignment | Reference |
| --- | --- | --- | --- | --- |
| 780 | 779 | C-H vibration | Lignin | Pancholi et al., 2022 |
| 829 | 833 | C-H stretching | Lignin | Sammons et al., 2013 |
| 875 | 869 | Glycosidic linkage | Hemicellulose | Xu et al., 2013 |
| 898 | 898 | C-H vibration | Cellulose | Ong et al., 2020 |
| 990 | 991 | C-O vibration | Cellulose | Xu et al., 2013 |
| 1163 | 1162 | C-O-C asymmetric stretching | Cellulose | Guo et al, 2016 |
| 1440 | 1442 | O-H bending | Cellulose, hemicellulose, Lignin | Xu et al., 2013 |

## Slide 4
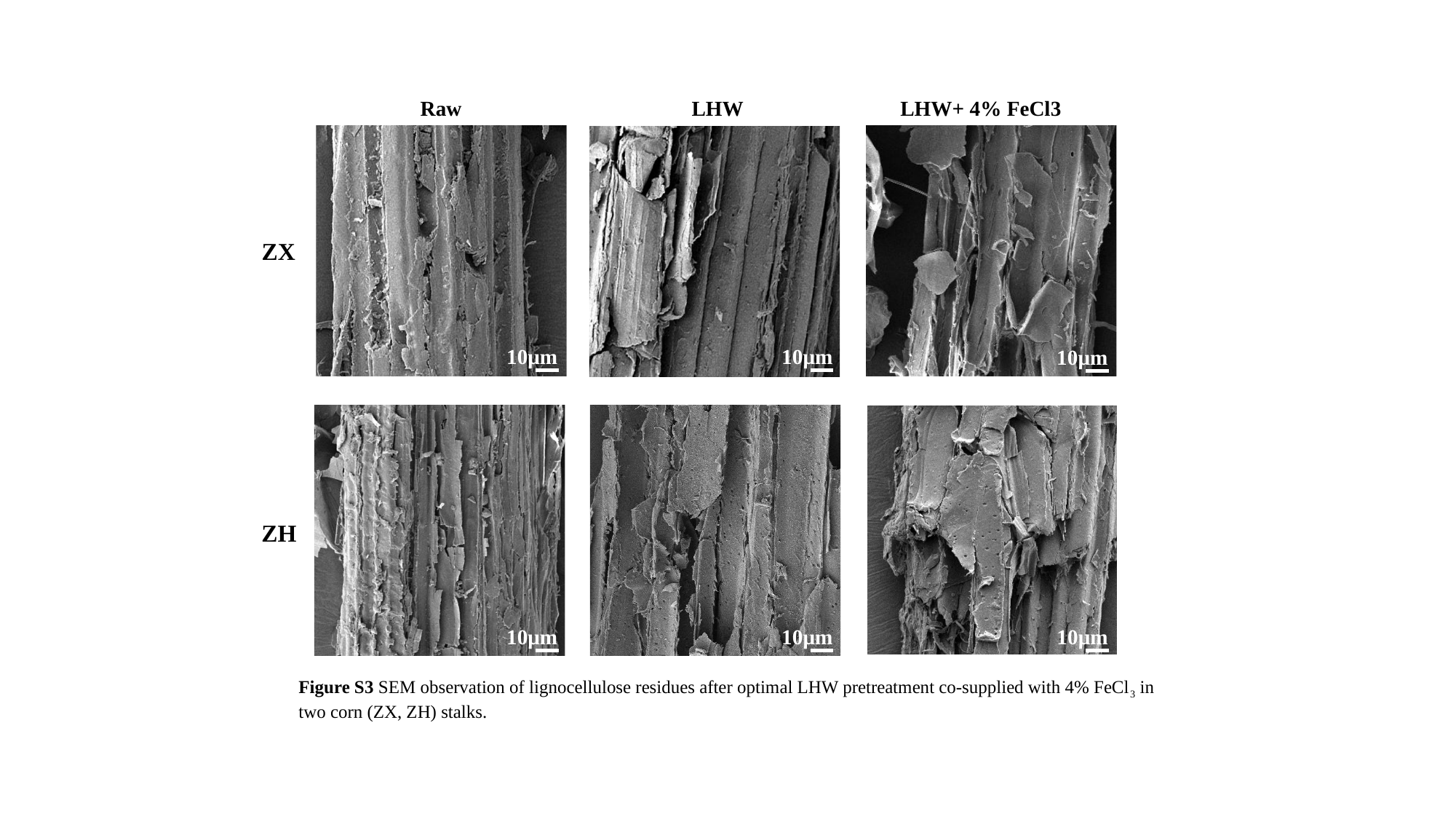

Raw
LHW
LHW+ 4% FeCl3
ZX
ZH
10μm
10μm
10μm
10μm
10μm
10μm
Figure S3 SEM observation of lignocellulose residues after optimal LHW pretreatment co-supplied with 4% FeCl3 in two corn (ZX, ZH) stalks.

## Slide 5
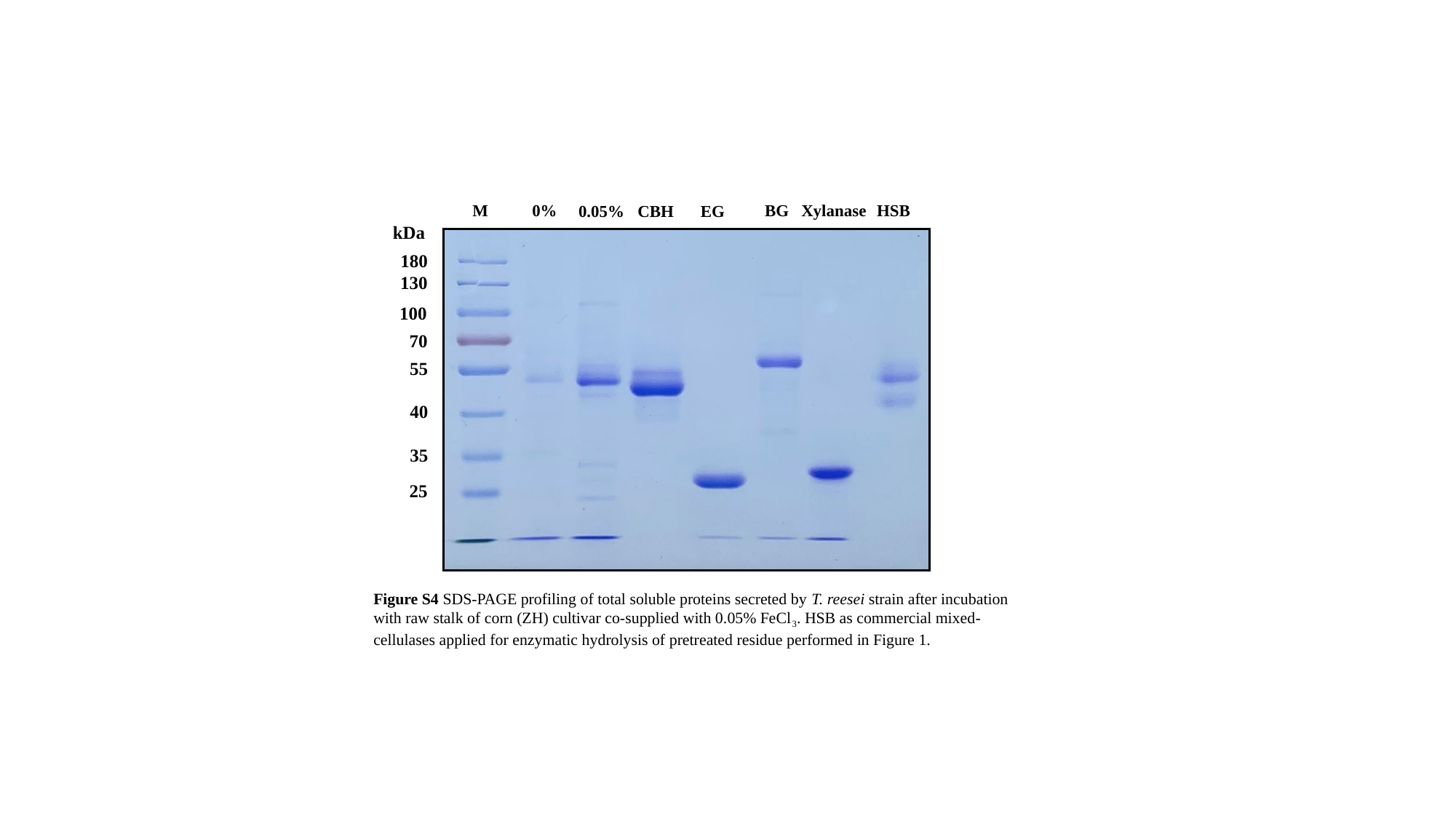

M
HSB
Xylanase
0%
BG
0.05%
EG
CBH
kDa
180
130
100
70
55
40
35
25
Figure S4 SDS-PAGE profiling of total soluble proteins secreted by T. reesei strain after incubation with raw stalk of corn (ZH) cultivar co-supplied with 0.05% FeCl3. HSB as commercial mixed-cellulases applied for enzymatic hydrolysis of pretreated residue performed in Figure 1.

## Slide 6
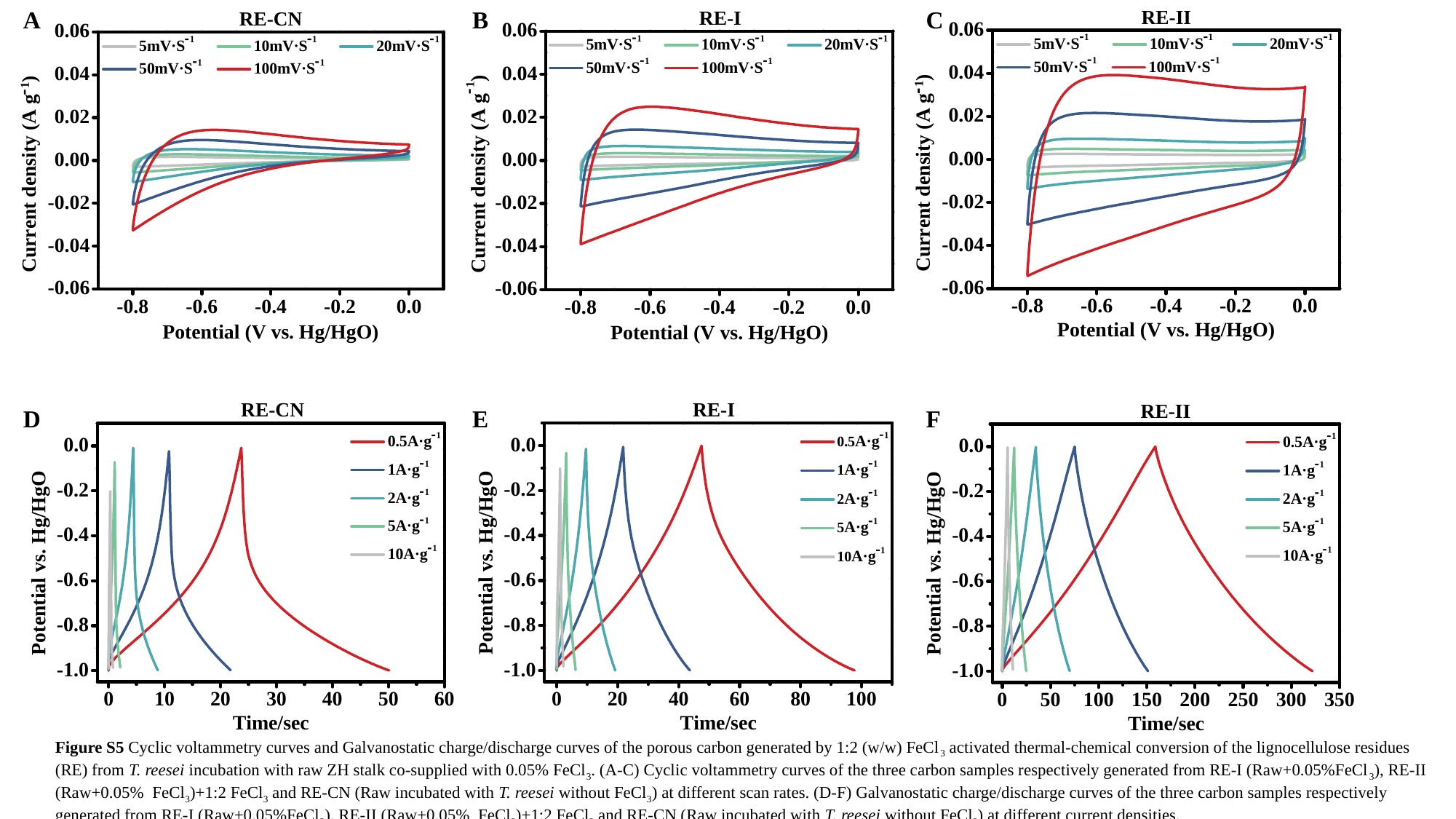

A
B
C
D
E
F
Figure S5 Cyclic voltammetry curves and Galvanostatic charge/discharge curves of the porous carbon generated by 1:2 (w/w) FeCl3 activated thermal-chemical conversion of the lignocellulose residues (RE) from T. reesei incubation with raw ZH stalk co-supplied with 0.05% FeCl3. (A-C) Cyclic voltammetry curves of the three carbon samples respectively generated from RE-I (Raw+0.05%FeCl3), RE-II (Raw+0.05% FeCl3)+1:2 FeCl3 and RE-CN (Raw incubated with T. reesei without FeCl3) at different scan rates. (D-F) Galvanostatic charge/discharge curves of the three carbon samples respectively generated from RE-I (Raw+0.05%FeCl3), RE-II (Raw+0.05% FeCl3)+1:2 FeCl3 and RE-CN (Raw incubated with T. reesei without FeCl3) at different current densities.
